# miR-153-3p suppresses the differentiation and proliferation of neural stem cells via targeting GPR55

**DOI:** 10.18632/aging.204002

**Published:** 2023-08-27

**Authors:** Xiaolin Dong, Hui Wang, Liping Zhan, Qingyun Li, Yang Li, Gang Wu, Huan Wei, Yanping Li

**Affiliations:** 1 Department of Neurology, The Affiliated Yan’an Hospital of Kunming Medical University, Kunming 650051, Yunnan, China; 2 Department of Gastroenterology, The Affiliated Yan’an Hospital of Kunming Medical University, Kunming 650051, Yunnan, China

**Keywords:** miR-153-3p, Alzheimer's disease, GPR55, neurodegenerative diseases

## Abstract

Alzheimer's disease is the most frequent neurodegenerative disease and is characterized by progressive cognitive impairment and decline. NSCs (neural stem cells) serve as beneficial and promising adjuncts to treat Alzheimer's disease. This study aimed to determine the role of miR-153-3p expression in NSC differentiation and proliferation. We illustrated that miR-153-3p was decreased and GPR55 was upregulated during NSC differentiation. IL-1β can induce miR-153-3p expression. Luciferase reporter analysis noted that elevated expression of miR-153-3p significantly inhibited the luciferase value of the WT reporter plasmid but did not change the luciferase value of the mut reporter plasmid. Ectopic miR-153-3p expression suppressed GPR55 expression in NSCs and identified GPR55 as a direct target gene of miR-153-3p. Ectopic expression of miR-153-3p inhibited NSC growth and differentiation into astrocytes and neurons. Elevated expression of miR-153-3p induced the release of proinflammatory cytokines, such as TNF-α, IL-1β and IL-6, in NSCs. Furthermore, miR-153-3p inhibited NSC differentiation and proliferation by targeting GPR55 expression. These data suggested that miR-153-3p may act as a clinical target for the therapeutics of neurodegenerative diseases.

## INTRODUCTION

AD (Alzheimer's disease) is the most frequent neurodegenerative disease and is characterized by progressive cognitive impairment and decline [1–4]. However, available therapeutic methods for AD are still lacking because of the unclear pathogenesis and etiology of this disease [5–7]. NSCs (neural stem cells) are multipotent and self-renewing cells found in the central nervous system of adults and developing mammals [8–10]. These stem cells differentiate into oligodendrocytes, astrocytes and neurons and serve as beneficial and promising adjuncts to treat neurological diseases such as spinal cord injuries, Parkinson’s disease, brain trauma and AD [11–14]. However, many challenges must be solved before the clinical use of NSCs.

MiRNAs are a family of 19- to 24-nucleotide noncoding endogenous RNAs that modulate gene expression at the posttranscriptional level by binding to the 3’-UTR of target mRNA [9, 15–18]. miRNAs are critical regulators of abundant biological processes such as apoptosis, differentiation, and chemoresistance [19–22]. Several miRNAs are regulated in many diseases, including AD, Parkinson’s disease, spinal cord injuries, brain trauma and tumors [23–27]. Recently, miRNAs were also reported to participate in the differentiation and proliferation of NSCs [12, 28, 29]. For example, Wu et al. [30]. illustrated that miR-374b modulated NSC differentiation and growth by regulating Hes1. Chen et al. [31]. noted that miR-132 acted as a moderator of neurite outgrowth, cell differentiation and self-renewal of NSCs. Recently, growing evidence has suggested that miR-153-3p induces immune dysregulation by suppressing PELI1 expression in MSCs (mesenchymal stem cells) that are separated from systemic lupus erythematosus patients [32]. However, the potential functional role of miR-153-3p in the fate of NSCs remains unclear.

In our study, we illustrated that miR-153-3p inhibited NSC differentiation and proliferation and proinflammatory cytokine release by targeting GPR55 expression in NSCs.

## MATERIALS AND METHODS

### Cell culture and transfection

NSCs were separated and cultured as described previously [33]. Cells were separated from five rat embryos and placed in medium supplemented with N2, bFGF and EGF. Our study was approved by The Affiliated Yan’An Hospital of Kunming Medical University. The miR-153-3p control, inhibitor and their control plasmids were obtained from GenePharma (China) and transfected into cells with Lipofectamine 3000 at a concentration of 10 nmol/l.

### qRT-PCR

Total RNA, including small RNA and mRNAs, was separated from NSCs using a TRIzol kit (Thermo Fisher, Inc., USA). The miRNA and mRNA levels were determined by RT-qPCR. RT-qPCR analysis was performed using a SYBR Premix kit (Takara, China) and the 7900HT system. U6 was used as an internal control for miRNA, and GAPDH was used as a control for mRNA. The 2^−ΔΔCT^ method was performed to determine the relative expression of target genes. The primer sequences were as follows: Tuj1, 5’-AGCAA GGTGC GTGAG GAGTA-3’ (forward) and 5’- AAGCC GGGCA TGAAG AAGT-3’ (reverse); Nestin 5’- GATCT AAACA GGAAG GAAAT CCAGG-3’ and 5’- TCTAGT GTCTC ATGGC TCTGGT TTT-3’; GFAP 5’-CAACG TTAAG CTAGC CCTGG ACAT-3’, and 5’-CTCAC CATCC CGCAT CTCCA CAGT-3’ and GAPDH 5’-ATTCC ATGGC ACCGT CAAGG CTGA-3’, and 5’-TTCTC CATGG TGGTG AAGAC GCCA-3’.

### Dual luciferase assay

The wild-type 3’-UTR and mutant 3’-UTR of GPR55 containing the predicted binding site of miR-153-3p were amplified by PCR and inserted into the pMIR-REPORT luciferase plasmid. Lipofectamine-2000 was utilized for transfection with miR-153-3p control or mimic and the wild-type and mutant 3’-UTRs of GPR55 as described previously. Luciferase activity was detected using a luciferase reporter kit (Promega, USA).

### ELISA

After treatment, the cell culture supernatant was obtained to measure the levels of proinflammatory cytokines such as TNF-α, IL-1β and IL-6 by using ELISA kits (Cambridge, UK).

### Proliferation assay

Cells were plated in 96-well dishes and were allowed to continue growing for 0, 1, 2 and 3 days after treatment. Cell growth was detected using Cell Counting Kit-8 (Dojindo, China), and the cells were incubated with CCK-8 reagent (10%) for 3 hours at 37° C. The absorbance was measured using a microplate reader at 450 nm.

### Immunofluorescence

Cells were fixed in paraformaldehyde (4%) for half an hour at room temperature and then washed in PBS (phosphate-buffered saline) 3 times. After 1 hour of blocking in Triton X-100 (0.2%) and goat serum (3%) in PBS, the cells were incubated with anti-nestin, anti-Tuj1 and anti-GFAP (1:400; Millipore, USA) at 4° C overnight. After washing 3 times in PBS, the cells were incubated with secondary antibodies. The cells were visualized with fluorescence microscope.

### Statistical analysis

Experimental statistics were presented as means±standard deviation. Statistical significance (P<0.05) was analyzed by ANOVA or Student's t-test using the SPSS software system (Chicago, USA).

## RESULTS

### NSC identification and culture

**Figure 1:**
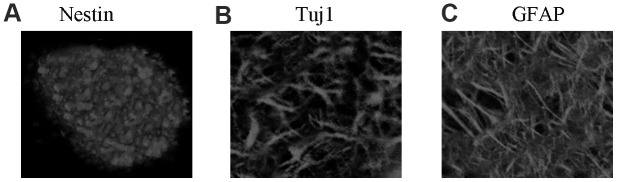
**NSC identification and culture.** (**A**) Immunocytochemical staining of purified neural stem cells with Nestin. (**B**) Immunocytochemical staining of neurons with Tuj1. (**C**) Immunocytochemical staining of astrocytes with GFAP.

Isolated rat NSCs self-proliferated and then formed several neurospheres, and the neurospheres expressed the NSC-specific marker nestin (Figure 1A). After removal of bFGF and EGF, NSCs were then differentiated into astrocytes and neurons. These rat cells expressed the neuron-specific marker Tuj1 (Figure 1B) and astrocyte marker GFAP (Figure 1C). Thus, the extracted NSCs were viable and suitable for further experiments.

### miR-153-3p is decreased and GPR55 is overexpressed during NSC differentiation

**Figure 2:**
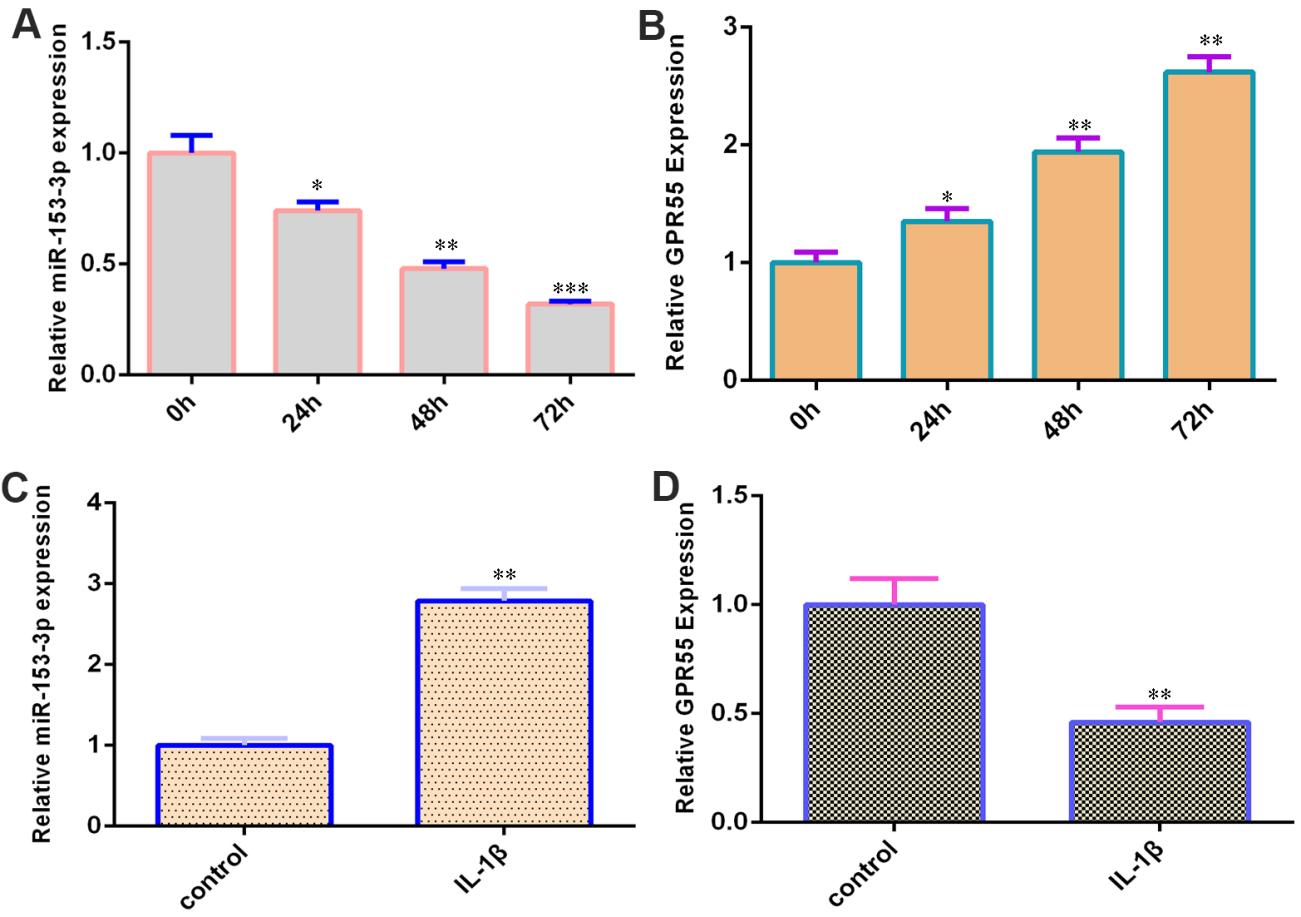
**miR-153-3p is decreased and GPR55 is overexpressed during NSC differentiation.** (**A**) The expression of miR-153-3p was measured by qRT-PCR. (**B**) The expression of GPR55 was measured by qRT-PCR. (**C**) IL-1β induces miR-153-3p expression in NSCs. (**D**) The expression of GPR55 is determined by qRT-PCR. *p<0.05, **p<0.01 and ***p<0.001. Error bars represent the s.d. of relative experiment from n=3 replicates.

qRT-PCR data showed that miR-153-3p was decreased during NSC differentiation (Figure 2A). Moreover, GPR55 was upregulated during NSC differentiation (Figure 2B). IL-1β (50 ng/ml) induced miR-153-3p expression in NSCs by two-fold (Figure 2C, p<0.01), and GPR55 was downregulated in NSCs after treatment with IL-1β compared with that in the control group by two-fold (Figure 2D, p<0.01).

### GPR55 is a direct gene target of miR-153-3p

**Figure 3:**
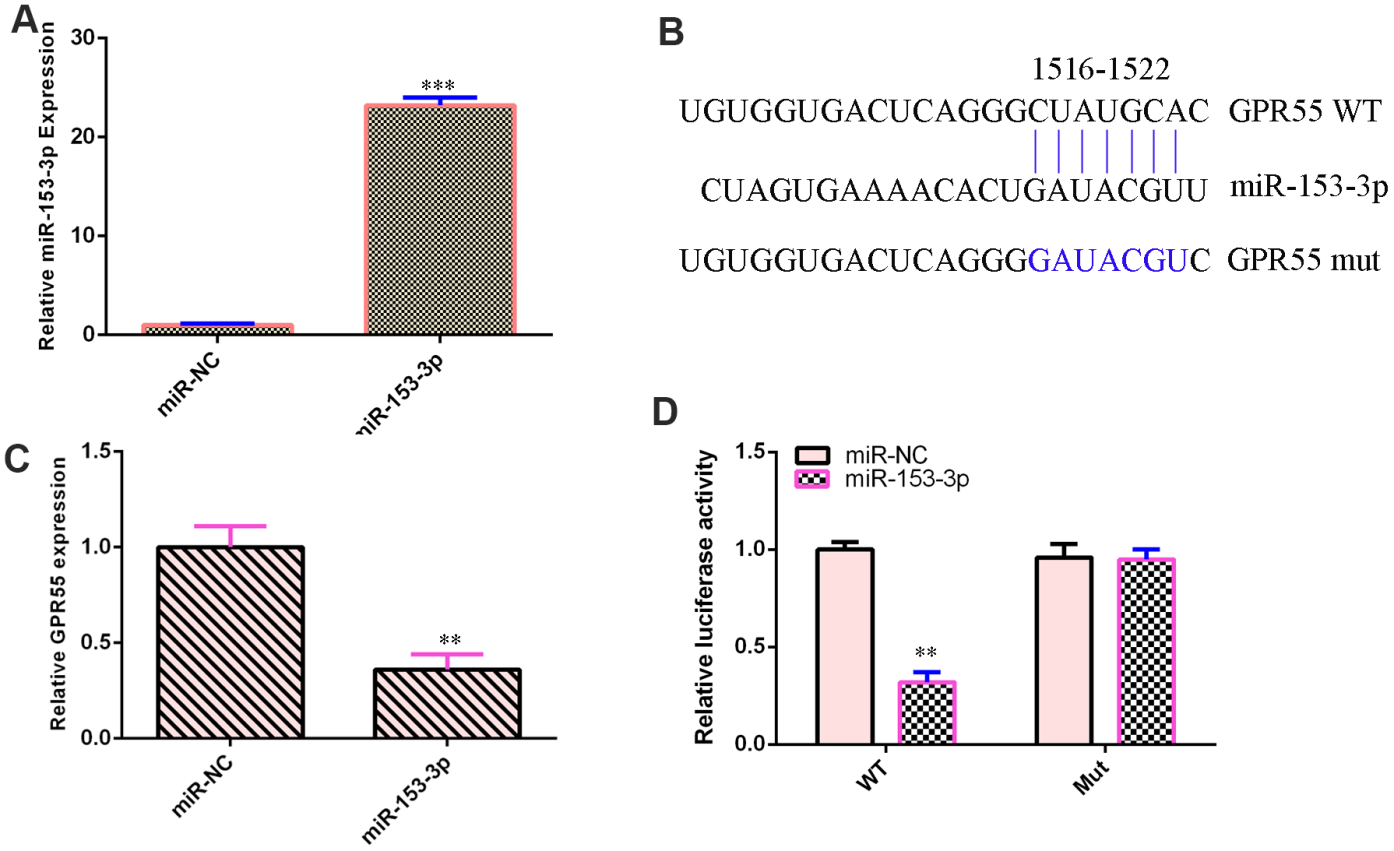
**GPR55 is a direct gene target of miR-153-3p.** (**A**) The expression of GPR55 was determined by qRT-PCR. (**B**) One potential target site was found between miR-153-3p and GPR55. These sequences were conserved between different species. (**C**) The expression of GPR55 was determined by qRT-PCR. (**D**) Luciferase reporter analysis noted that elevated expression of miR-153-3p significantly inhibited the luciferase value of the WT reporter plasmid but did not change the luciferase value of the mut reporter plasmid. **p<0.01 and ***p<0.001. Error bars represent the s.d. of relative experiment from n=3 replicates.

qPCR illustrated that miR-153-3p was overexpressed in NSCs after treatment with the miR-153-3p mimic compared with that in the miR-NC group (Figure 3A, p<0.001). This result suggested that the efficiency of miR-153-3p was high. By searching bioinformatic TargetScan 7.2 (http://www.targetscan.org/vert_72/), we identified one potential target site between miR-153-3p and the GPR55 3’-UTR (Figure 3B). We also showed that these sequences were conserved among different species (Figure 3B). Ectopic miR-153-3p expression suppressed GPR55 expression in NSCs (Figure 3C, p<0.01). Luciferase reporter analysis noted that elevated expression of miR-153-3p significantly inhibited the luciferase value of the WT reporter plasmid but did not change the luciferase value of the mut reporter plasmid (Figure 3D, p<0.01).

### miR-153-3p suppresses NSC differentiation and proliferation

**Figure 4:**
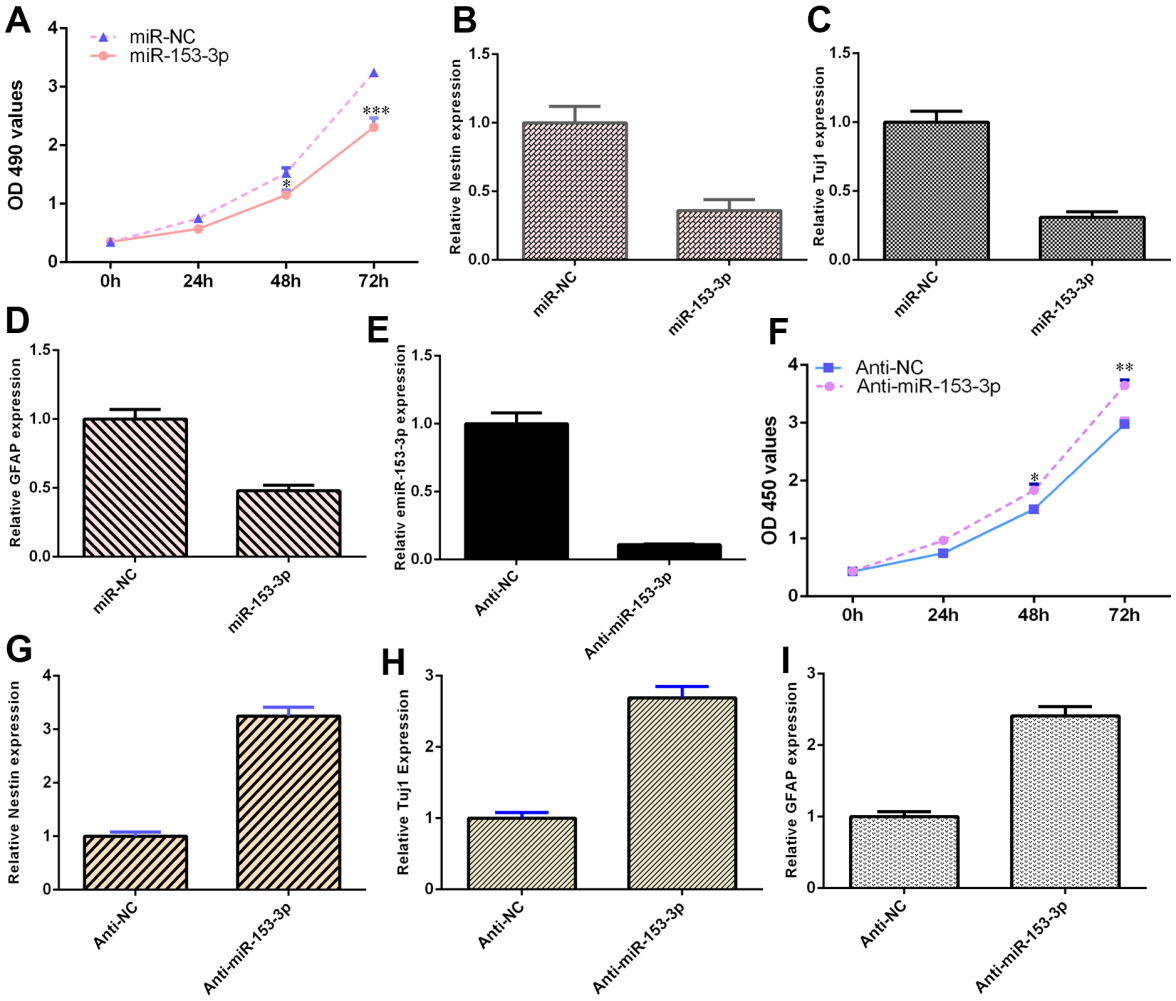
**miR-153-3p suppresses NSC differentiation and proliferation.** (**A**) Ectopic expression of miR-153-3p inhibited NSC proliferation. (**B**) Overexpression of miR-153-3p decreased nestin expression. (**C**) The expression of Tuj1 was detected by qRT-PCR. (**D**) The expression of GFAP was measured by qRT-PCR. (**E**) The expression of miR-153-3p was measured by qRT-PCR. (**F**) The suppression of miR-153-3p increased NSC growth. (**G**) Nestin expression was measured by qRT-PCR. (**H**) The expression of Tuj1 was detected by qRT-PCR. (**I**) The expression of GFAP was measured by qRT-PCR. *p<0.05, **p<0.01 and ***p<0.001. Error bars represent the s.d. of relative experiment from n=3 replicates.

The expression of miR-153-3p was downregulated in NSCs after treatment with the anti-miR-153-3p mimic (Figure 4E, p<0.001). Ectopic expression of miR-153-3p inhibited NSC proliferation (Figure 4A, p<0.001), and miR-153-3p suppression increased NSC growth (Figure 4F, p<0.01). Additionally, miR-153-3p overexpression decreased nestin expression (Figure 4B, p<0.01) and miR-153-3p knockdown induced nestin expression (Figure 4G, p<0.01) in NSCs. Furthermore, we illustrated that ectopic miR-153-3p expression suppressed Tuj1 (Figure 4C, p<0.01) and GFAP (Figure 4D, p<0.01) and that miR-153-3p suppression enhanced Tuj1 (Figure 4H, p<0.01) and GFAP (Figure 4I, p<0.01) in NSCs. Taken together, these data showed that miR-153-3p inhibited cell differentiation and proliferation in NSCs.

### miR-153-3p induces proinflammatory cytokine release

**Figure 5:**
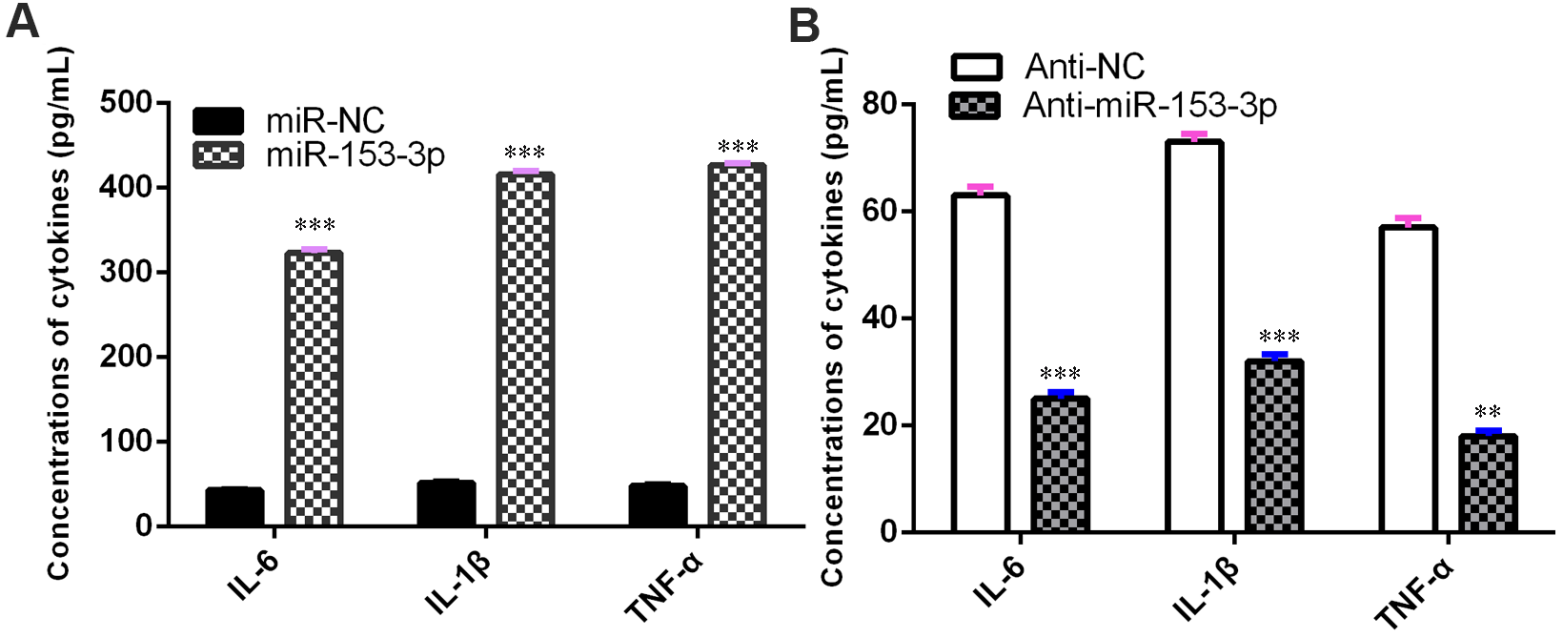
**miR-153-3p induces proinflammatory cytokine release.** (**A**) The concentration levels of TNF-α, IL-1β and IL-6 were upregulated in NSCs after treatment with the miR-153-3p mimic. (**B**) Knockdown of miR-153-3p suppressed the concentration levels of TNF-α, IL-1β and IL-6 in NSCs. **p<0.01 and ***p<0.001. Error bars represent the s.d. of relative experiment from n=3 replicates.

The concentrations of TNF-α, IL-1β and IL-6 were upregulated in NSCs after treatment with the miR-153-3p mimic (Figure 5A, p<0.001). However, the knockdown of miR-153-3p suppressed the levels of TNF-α, IL-1β and IL-6 in NSCs (Figure 5B, p<0.01). These data suggested that miR-153-3p induced proinflammatory cytokine release.

### miR-153-3p inhibits NSC differentiation and proliferation by targeting GPR55 expression

**Figure 6:**
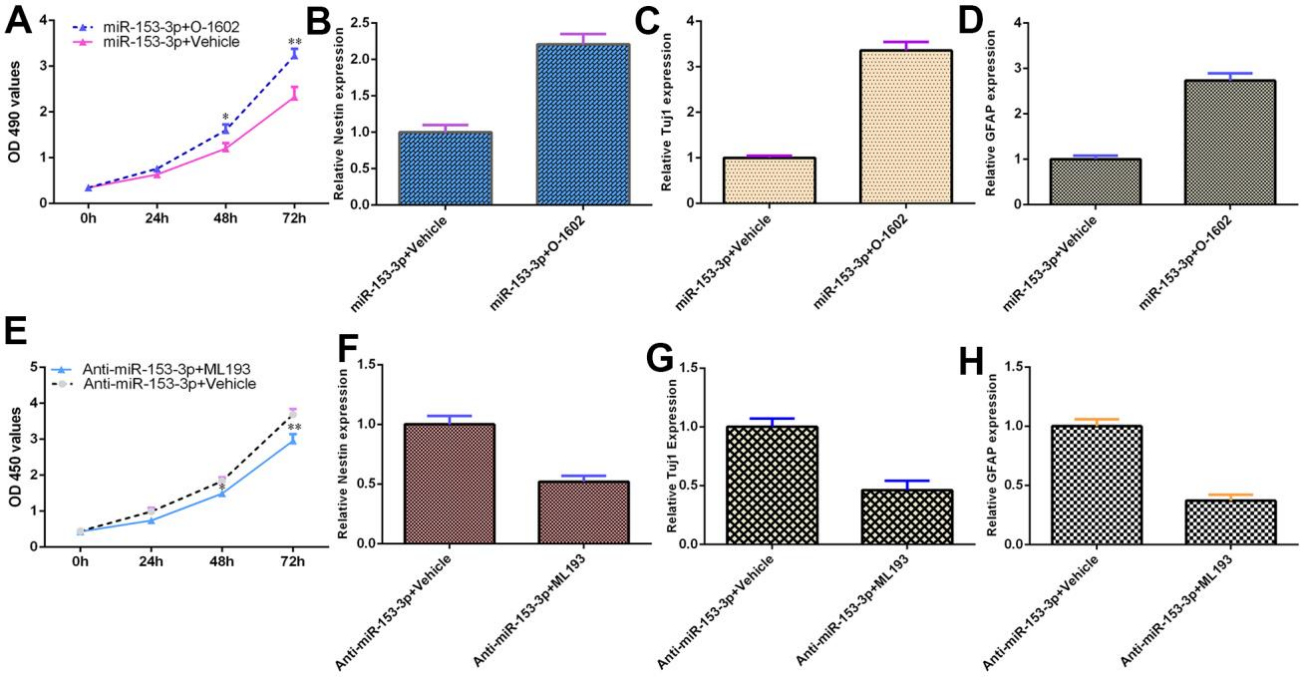
**miR-153-3p inhibits NSC differentiation and proliferation by targeting GPR55 expression.** (**A**) Cell proliferation was measured using CCK-8 analysis. (**B**) Nestin expression was determined by qRT-PCR. (**C**) The expression of Tuj1 was detected by qRT-PCR. (**D**) The expression of GFAP was measured by qRT-PCR. (**E**) The GPR55 antagonist ML-193 inhibited cell growth compared with the vehicle group in miR-153-3p-treated NSCs. (**F**) Nestin expression was determined by qRT-PCR analysis. (**G**) The expression of Tuj1 was detected by qRT-PCR. (**H**) The expression of GFAP was measured by qRT-PCR. *p<0.05 and **p<0.01. Error bars represent the s.d. of relative experiment from n=3 replicates.

Because GPR55 is an NSC regulator, we speculated that miR-153-3p might act on these functions by regulating GPR55 expression. To prove this hypothesis, several gain and loss function experiments were performed. The GPR55 agonist O-1602 promoted cell proliferation compared with the vehicle group (Figure 6A, p<0.01), and the GPR55 antagonist ML-193 inhibited cell growth compared with the vehicle group (Figure 6F, p<0.01) in miR-153-3p-treated NSCs. The GPR55 agonist O-1602 increased nestin (Figure 6B, p<0.05), Tuj1 (Figure 6C, p<0.05) and GFAP (Figure 6D, p<0.05) expression compared with the vehicle group, and the GPR55 antagonist ML-193 decreased nestin (Figure 6G, p<0.05), Tuj1 (Figure 6H, p<0.05) and GFAP (Figure 6I, p<0.05) expression compared with the vehicle group in miR-153-3p-treated NSCs. These data were also confirmed using immunocytochemical staining (Figure 6E). These results showed that miR-153-3p inhibited NSC differentiation and proliferation by targeting GPR55 expression.

## DISCUSSION

Previous studies have illustrated that miRNAs regulate NSC differentiation, neuronal maturation and proliferation [10, 34, 35]. For example, Wu et al. [30]. illustrated that miR-374b modulates NSC differentiation and growth by regulating Hes1. Chen et al. [31]. noted that miR-132 acts as a moderator of neurite outgrowth, cell differentiation and self-renewal of NSCs. Xue et al. [36]. indicated that miR-145 protects NSC function by regulating the MAPK signaling pathway to remediate rat cerebral ischemic stroke. Channakkar et al. [37]. showed that miR-137 modulates NSC fate via the regulation of mitochondrial dynamics. However, the potential functional role of miR-153-3p in the fate of NSCs remains unclear. miR-153-3p modulates cisplatin resistance and cell growth through Nrf-2 in esophageal carcinoma [38]. Li et al. [39]. illustrated that miR-153-3p modulates ovarian carcinoma progression by regulating MCL1 expression. Sun et al. [40]. indicated that miR-153-3p promotes glioma cell radiosensitivity by modulating BCL2. A previous study showed that IL-1β induces miR-153 expression in beta cells and that IL-1β plays critical roles in the fate of NSCs [41–43]. In the present study, we noted that miR-153-3p is decreased during NSC differentiation and that IL-1β induces miR-153-3p expression in NSCs. Ectopic expression of miR-153-3p inhibited NSC growth and differentiation into astrocytes and neurons. Elevated expression of miR-153-3p induced the release of proinflammatory cytokines, such as TNF-α, IL-1β and IL-6, in NSCs. These results indicated that miR-153-3p plays critical roles in the cell differentiation and self-renewal of NSCs.

GPR55 is a lipid-sensing receptor that plays important roles in cell mobilization, invasion and cell cycle progression in tumor development. Wang et al. [44]. indicated that CID16020046 (GPR55 antagonist) protects against inflammation induced by ox-LDL in HAECs (aortic endothelial cells). Saliba et al. [45]. illustrated that several compounds with antagonistic activities of GPR55 suppress PGE2 release in microglia. Recently, Hill et al. [46]. showed that GPR55 activation promotes NSC proliferation and differentiation into neuronal cells. Moreover, they found that a GPR55 agonist defends against neurogenesis rate reductions in NSCs induced by IL-1β. GPR55 activation suppresses inflammatory cytokine expression in NSCs [47]. In our study, we searched for bioinformatic targets and identified one potential target site between miR-153-3p and the GPR55 3’-UTR. Luciferase reporter analysis noted that the elevated expression of miR-153-3p significantly inhibited the luciferase value of the WT reporter plasmid but did not change the luciferase value of the mut reporter plasmid. Moreover, we showed that ectopic expression of miR-153-3p suppresses GPR55 expression in NSCs. Furthermore, miR-153-3p inhibited NSC differentiation and proliferation by targeting GPR55 expression. However, more experiments must be performed on human NSCs in the future. These results provide novel insights into the modulation of GPR55 and its cell function in the development of NSCs.

In summary, our results noted the involvement of miR-153-3p in modulating the differentiation and growth of NSCs. It also illustrated that miR-153-3p inhibits NSC differentiation and proliferation and proinflammatory cytokine release by targeting GPR55 expression in NSCs. These data suggest that miR-153-3p acts as a clinical target for neurodegenerative disease therapeutics.
